# Sensing Technology for Assessing Motor Behavior in Ballet: A Systematic Review

**DOI:** 10.1186/s40798-022-00429-8

**Published:** 2022-03-14

**Authors:** Virginia Quadrado, Margarida Moreira, Hugo Ferreira, Pedro Passos

**Affiliations:** 1 CIPER, Faculdade de Motricidade Humana, Universidade de Lisboa, Estrada da Costa, 1499-002 Lisbon, Portugal; 2 Instituto de Biofísica e Engenharia Biomédica, Faculdade de Ciências da Universidade de Lisboa, 1749-016 Lisbon, Portugal

**Keywords:** Sensing technology, Motor behavior, Human performance, Ballet, Dance

## Abstract

**Background:**

Human performance in classical ballet is a research field of growing interest in the past decades. Technology used to acquire data in human movement sciences has evolved, and is specifically being applied to evaluate ballet movements to better understand dancers’ profiles. We aimed to systematically review sensing technologies that were used to extract data from dancers, in order to improve knowledge regarding the performance of ballet movements through quantification.

**Methods:**

PubMed, MEDLINE, EMBASE, and Web of Science databases were accessed through 2020. All studies that used motor control tools to evaluate classical ballet movements, and possible comparisons to other types of dance and sports movements were selected. Pertinent data were filled into a customized table, and risk of bias was carefully analyzed.

**Results:**

Eighty studies were included. The majority were regarding classical ballet and with pre-professional dancers. Forty-four studies (55%) used two or more types of technology to collect data, showing that motion capture technique, force plates, electromyography, and inertial sensors are the most frequent ways to evaluate ballet movements.

**Discussion:**

Research to evaluate ballet movements varies greatly considering study design and specific intervention characteristics. Combining two or more types of technology may increase data reliability and optimize the characterization of ballet movements. A lack of studies addressing muscle–brain interaction in dancers were observed, and given the potential of novel insights, further studies in this field are warranted. Finally, using quantitative tools opens the perspective of defining what is considered an elite dancer.

## Background

Motor behavior in dance has been a field of growing interest in the past decades. In particular, since the early 1960s, literature shows research approaches regarding movement performance of the human body from the dance perspective [1].

In 2009, a literature review was published regarding biomechanics measurement tools used in dance [2]. The authors reviewed and analyzed studies concerning selected ballet movements, measurement tools, research design, participants’ characteristics, and type of study. In the meantime, the number of studies in the past ten years has substantially increased, not only considering the increased demand for dance research, but especially due to the evolution of digital technologies that have allowed researchers to collect exponentially more data with unprecedented accuracy. Thus, the present systematic review aims to update the literature with all the findings made throughout the years regarding studies in motor behavior in ballet, especially focusing on the digital sensing technologies used. This systematic review offers then not only an updated description concerning measurement tools and data collection in dance, but also the ballet movements of interest and trends of study, identifying future potential avenues for research.

For additional context, several literatures and systematic reviews have been published in the past decade on the topic of classical ballet, but mostly addressing issues such as injuries and rehabilitation processes [3–6], finding and compiling techniques that may help dancers to prevent injuries or to recover from them. However, four systematic reviews were found regarding motor behavior and biomechanics analysis associated with dance [2, 7–9]. By studying isolated parts of the body or analyzing a specific movement, researchers reviewed studies in order to understand what has been explored in the dance field and what is still to be discovered. Herein, the present systematic review aims instead to explore which digital sensing technologies have been used to capture data specifically from ballet movements. Finally, ballet research has also captured the interest of neuroscientists, aiming to understand the brain mechanisms involved in dance, as well as the mechanisms that could possibly differentiate elite dancers from novices, through systematic reviews that analyzed mental imagery and cortical activity during imagery tasks [10–12]. In the present review only those digital technologies addressing these latter topics were the object of our research.

## Methods

This systematic review conforms to the Preferred Reporting Items for Systematic Reviews and Meta-Analysis (PRISMA) statement [13] and has been registered in the International Prospective Register of Systematic Reviews (PROSPERO, protocol no. CRD42020206680) [14].

Four database search engines (PubMed, MEDLINE, EMBASE, and Web of Science) were used to identify eligible scientific articles regarding human performance and motor behavior in ballet and dance (i.e., contemporary dance and modern dance), sensing technology, and instruments and tools for data capture in dance. The search encompassed literature published until December 2020, with headings and keywords related to motor behavior in ballet ((classical ballet OR dancing OR elite dancers) AND (randomized controlled trials OR RCT OR quasi-RCT); (classical ballet OR classical dancing OR classical dance OR ballet OR elite dancers) AND (biomechanics OR biomechanical tools OR biomechanics instruments OR biomechanics analysis); (ballet movements OR ballet positions OR dance movements OR elite dancers) AND (measurement tools OR sensing technology OR motor behavior OR human performance); (EMG OR sEMG OR electromyography OR surface electromyography OR muscle activity) AND (classical ballet OR classical dance OR classical dancing OR ballet movement OR dance movement OR elite dancers); (GRF OR ground force reaction OR kinetic analysis) AND (classical ballet OR classical dance OR classical dancing OR ballet movement OR dance movement OR elite dancers); (motion capture OR kinematic analysis OR motion analysis) AND (classical ballet OR classical dance OR classical dancing OR ballet movement OR dance movement OR elite dancers); (accelerometer OR inertial sensor OR inertial sensors) AND (classical ballet OR classical dance OR classical dancing OR ballet movement OR dance movement OR elite dancers); (EEG OR electroencephalography) AND (classical ballet OR classical dance OR classical dancing OR ballet movement OR dance movement OR elite dancers)), and disregarding articles related to injury evaluation, rehabilitation purposes, and neurological disorders.

### Inclusion and Exclusion Criteria

Inclusion criteria were defined by type of dance, participants, and research tools. Studies that evaluated classical ballet movements and possible comparisons to other types of dance and sports were included. Participants of those studies were regarded as classical, modern, and contemporary dancers. Articles involving tools such as 3D cameras, motion capture, laser sensors, video analysis, cinematography analysis, inverse dynamic analysis, image reconstruction, force plates, seesaw plates, dynamometers, accelerometers, inertial sensors, and surface EMG (sEMG) were included in our search. We considered studies without language restrictions; however, all the selected articles were published in English.

As exclusion criteria, articles containing only abstract, conference proceedings, systematic reviews, and other types of literature review and studies conducted involving older adults and with purposes of rehabilitation treatment were excluded. Articles involving manual measurement through analog tools (i.e., goniometers and/or measurement tapes), magnetic resonance imaging (MRI), X-rays, and ultrasound as isolated techniques of analysis were also excluded.

### Data Management

One of the authors screened the titles and abstracts of all identified studies according to the selection criteria. Full texts were then retrieved. Two other authors independently extracted the data and reached consensus, filling a designed table to extract pertinent data. The ROBINS scale [15] was applied to analyze risk of bias, because most of the retrieved articles were non-randomized controlled trials (RCT). For the RCT studies, risk of bias was analyzed through the Cochrane Collaboration’s tool [16].

## Results

### Literature Search

**Fig. 1:**
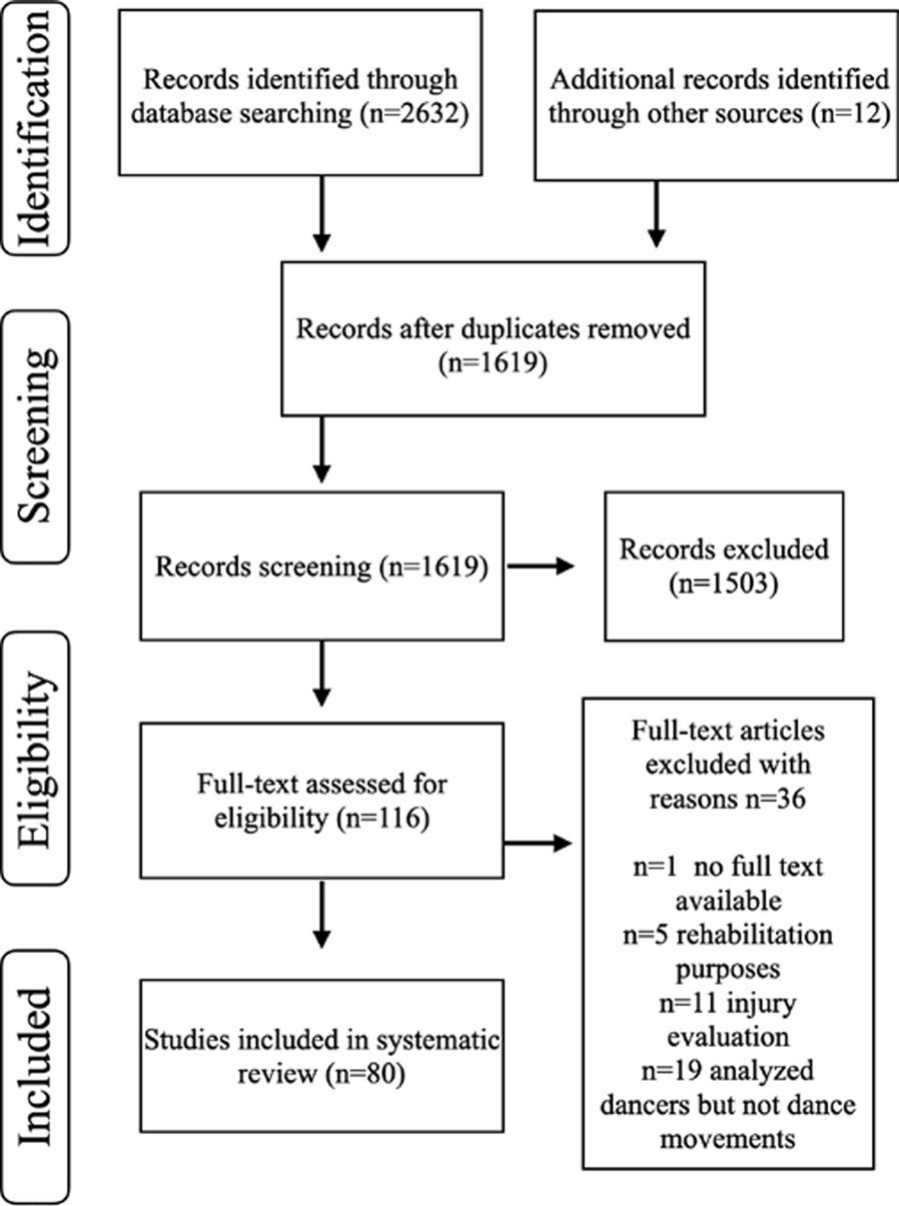
Diagram of information through the different phases in the systematic review

The database search process retrieved 2632 potentially relevant articles. References of the included articles were then scanned to ensure that relevant literature was not excluded from the review, and 12 additional records were identified. After duplicates were removed, the number of articles decreased to 1619. Articles were screened first by title and abstract for relevance to ballet, motor control sensing technology tools, and finally by full text (*n* = 116 full texts were assessed for eligibility) using the inclusion and exclusion criteria. After the evaluation process, 80 studies met the inclusion criteria. Articles were not limited by year of publication; however, the earliest article found regarding our search terms was published in 1993. We included articles published throughout the years until December 2020 (Fig. 1).

### Quality Index

Regarding the 80 studies included in the present systematic review, only 3 studies were RCTs, and their risk of bias was analyzed through the Cochrane Collaboration’s tool for assessing risk of bias [16–18]. The 3 studies showed the same outcome, as high risk in 4 out of the 7 analyzed variables as described “random sequence generation”, “allocation concealment”; “blinding of participants and personnel”; “blinding of outcome assessment”, and low risk for the variables “incomplete outcome data”; “selective reporting”, and “other sources of bias”. The remaining 77 studies were then analyzed through the ROBINS scale [15], and the obtained scores were 3 studies presenting low risk of bias, 37 studies low to moderate, 21 studies moderate, 8 studies moderate to serious, and 8 studies presenting serious risk of bias. Please see Table 1 for a detailed description.

**Table 1:** Participants characteristics, sensing technologies, category of movement, and risk of bias obtained from the studies included in this review

References	Participants	Category of dance	Sensing Technology	Ballet movement	Comparison between groups	Risk of bias
Weighart et al. [19]	Pre-professional; Age: 20 .3 ± 1.4; H/w: 16.4 ± 9.0 Female	Classical ballet and modern dance	Isokinetic dynamometer; EMG: VMO, VL (right leg)	Demi-plié & sauté in CP1, CP6	Ballet versus modern / injured versus non-injured	Low to moderate
Lott and Xu [20]	Pre-professional and Elite; years of training: 20 Female	Classical ballet	Motion capture	En dehors pirouette in CP4	–	Low to moderate
Arnwine and Powell [21]	Pre-professional and Elite; Age: F—23.4 ± 4.7, M—27.4 ± 4.4 Males and Females	Classical ballet	Force plates	Grand-jeté & sautés in CP1	Male versus female	Low to moderate
Jarvis et al. [22]	Elite; Age: 27.04 ± 3.99; Years of training: 21.07 ± 4.88 Female	Classical ballet	Motion capture; force plates	Saut de chat from chassé	–	Low to moderate
Skopal et al. [23]	Pre-professional; Age: 19 ± 2; Years of training: 9 ± 5.3; H/w: 12 ± 12.5 Female	Contemporary dance	Motion capture; Isokinetic dynamometer	Grand-jeté	Dancers (extra-training) versus dancers (regular training)	Low to moderate
Gorwa et al. [24]	Pre-professional; Age: Greater—13.9 ± 1.7, Lesser—15.1 ± 0.7; Years of training: Greater—4.1 ± 1.5, Lesser—5.6 ± 0.5 Female	Classical ballet	Motion capture; EMG: ES. RA, GM, SAR, BF, SEM, ADL, RF, VL, VM, LGAS, MGAS, TA, FIB	CP1 to 6	Greater hip turnout versus lesser hip turnout	Low to moderate
Seki et al. [25]	Pre-professional; Age: 20; Years of training: 10 Female	Classical ballet	Motion capture	Demi-plié with hallux valgus in CP1	–	Low to moderate
Greenwell et al. [26]	Pre-professional; Age: 20.5 Males and Females	Classical ballet and modern dance	Motion capture	Grand-plié in CP1, CP5	–	Moderate to serious
Hendry et al. [27]	Pre-professional; Age: 19.6 ± 1.2; H/w: At least 8 Female	Classical ballet and modern dance	Video analysis; Force plates; Inertial sensors	Sauté bilateral & unilateral	–	Low to moderate
Janura et al. [28]	Elite; Age: F: 25.6 ± 3.8 M: 23.4 ± 4.0; Years of training: At least 10; H/w: 3 to 8 Males and Females	Classical ballet	Force plates	Postural sway	Elite versus non-dancers	Moderate
Gorwa et al. [29]	Elite; Age: 28.6; Years of training: At least 9 Males and Females	Classical ballet and modern dance	Motion capture; Force plates	Grand-jeté, Entrelacé & Ballonné	–	Serious
Perry et al. [30]	Elite; Age: 20.7 ± 2.7; Years of training: 13.9 ± 5.0; Female	Classical ballet	Motion capture; Force plates	Saut de chat & temps levé	–	Low to moderate
Lin et al. [31]	Elite and Novices; Age: 17.8 ± 3.4; years of training: for novices, 2–5 years and for advanced at least 6 years; H/w: 1.5–3 h for novices, at least 3 h for advanced; Female	Classical ballet	Motion capture; Force plates	En dehors pirouette in CP4	Elite versus Novice	Moderate to serious
Lott [32]	Pre-professional; Age: 16 ± 1.4; Female	Classical ballet	Video analysis; Inverse dynamics	En dehors pirouette in CP4	–	Low
Blanco et al. [33]	Elite and pre-professional and Novices; Age: 20.1 ± 3.6; years of training: Novices: 1.3 ± 0.9 Pre-professional: 3.4 ± 1.4 Elite:7.2 ± 2.4; Males and Females	Classical ballet	Motion capture; force plates; Inertial sensors	Grand-jeté	Elite versus pre-professional versus novice	Serious
Carter et al. [34]	Pre-professional; Age: 18.8 ± 1.6; Years of training: 12.6 ± 3.6; H/w: 19.5 ± 8.8; Female	Classical ballet and modern dance	Motion capture	Turnout of CP1 & sauté in CP1	–	Low to moderate
Aquino et al. [35]	Elite; Age: 22.2 ± 2.2; Years of training: At least 10; Female	Classical ballet	Motion capture; Force plates; EMG: TA, MGAS (left leg)	Relevé in CP2 & piqué arabesque in CP4	–	Low to moderate
Mira et al. [36]	Elite and Novices; Age: Novices—16.7 ± 0.7, Elite—23.5 ± 1.5	Classical ballet	Motion capture; Force plates; EMG: MGAS, LGAS, SOL, VL	Cou-de-pied derrière with demi-plié to piqué arabesque	Elite versus Novice dancers	Moderate
McPherson et al. [37]	Elite; Age: 19.28 ± 1; Years of training: 12.85 ± 2.37; H/w: 15.02 ± 7.49; Female	Classical ballet	Video analysis; Force plates	Grand-jeté & assemblé dessus from tendu devant	–	Low
Imura and Iino [38]	Elite; Age: F—1935, M—28.3; Years of training: F—14, M—19.8; Males and Females	Classical ballet	Motion capture; Force plates	En dehors pirouette in CP4	–	Low to moderate
Bruyneel et al. [39]	Pre-professional; Age: Young—12.6 ± 1.95, Adult—22.4 ± 5.06; Years of training: at least 4; H/w: Young—14.4 h ± 8.49, Adult—23.8 h ± 10.61; Males and Females	Classical ballet	Force Plates	Grand-plié in CP1	Young with adult dancers	Moderate
Bickle et al. [40]	Elite; Age: 26 ± 4; Years of training: at least 3; H/w: at least 25; Female	Classical ballet	Video analysis; Force plates	Bourrés	Worn pointe shoes versus new pointe shoes	RCT High Risk
Michalska et al. [41]	Elite; Age:28. ± 7; Years of training; at least 5 with an average of 17; Female	Classical ballet	Force plates	Postural sway	Dancers versus non-dancers	Moderate
Carter et al. [42]	Pre-professional; Age: 19.1 ± 1.8; Years of training: 12.7 ± 3.9; H/w: 19.9 ± 9.7; Female	Classical ballet and modern dance	Motion capture	Turnout of CP1 & sautés in CP1	–	Moderate
Carter et al. [43]	Pre-professional; Age: 18.8 ± 0.8; Years of training: 11.7 ± 3.1; Female	Classical ballet and modern dance	Motion capture	Demi-plié & elevé in CP1, degagé devant (flex-point-flex)	–	Moderate
Costa de Mello et al. [44]	Elite; Age: 28.4 ± 10.8; Males and Females	Classical ballet	Force plates	Postural sway & retiré passé	Elite versus non-dancers	Moderate
Saito et al. [45]	Pre-professional; Age: 20.3 ± 1.6; Years of training: 16.8; H/w: 7.6; Female	Classical ballet	Force plates; EMG: SOL, MGAS	Elevé in CP6	Dancers versus non-dancers	Low to moderate
Imura and Iino [46]	Elite; Age: 30 ± 1; Female	Classical ballet	Motion capture; Force plates	Sauté in CP1, CP6	–	Low to moderate
Jarvis and Kulig [47]	Elite; Age: 27 ± 3.9; Years of training: 20.8 ± 4.9; Female	Classical ballet	Motion capture; Force plates	Saut de chat	–	Moderate
Hinton-Lewis et al. [48]	Pre-professional; Age: 19.2 ± 1.3; Years of training: F—5.2 ± 4.1, M—13.5 ± 3.3; Males and Females	Classical ballet	Video analysis; Inertial sensors	Demi-plié, relevé & sauté in CP1	Male versus Female	Moderate
Hopper et al. [49]	Pre-professional; Age: 19.2 ± 1.3; Years of training: at least 5; Males and Females	Classical ballet	Motion capture	Demi-plié, battement fondu with elevé & relevé, ballonné en place, ballonné traveling & Sissonne fondu	–	Moderate
Quanbeck et al. [50]	Pre-professional and Elite; Age: 20.3 ± 1.5; Years of training: 14.7 ± 2.5; Female	Classical ballet and modern dance	Motion capture	Turnout of CP1	–	Low to moderate
Brown and Meulenbroek [51]	Pre-professional; Age: 19.71 ± 2.09; Males and Females	Classical ballet	Inertial sensors	Port de bras in bras-bas, 1st, 2nd, 3rd, 3rd reversed, 1st, bras-bas, demi seconde allongé & bras-bas	–	Low to moderate
Steinberg et al. [52]	Elite; Age: F—16.67 ± 1.79, M—15.90 ± 1.42; Males and Females	Classical ballet	Inertial sensors	Postural sway in coud-de-pied and fondu	Male versus Female	Moderate to serious
Abraham et al. [53]	Elite; Age: 31 ± 1.87; Years of training: 22.8 ± 4.14; Males and Females	Contemporary dance	Motion capture	Elevé	–	Serious
Coker et al. [54]	Elite; Age: 26.04 ± 5.29; Years of training: 19.63 ± 6.47; Female	Classical ballet	Motion capture; Force plates	Demi-plié & sauté in CP1	VI and KI versus Mental arithmetic task as control group	RCT High Risk
Jarvis and Kulig [55]	Elite; Age: Between 18 and 35; Years of training: At least 10; Female	Classical ballet and modern dance	Motion capture; Force plates	Relevé, sauté & saut de chat in CP1	Elite versus non-dancers	Moderate
Bronner and Shippen [56]	Pre-professional and Elite; Age: Elite—25.8 ± 2.6, Pre-professional—20.4 ± 1.5; Years of training: Elite—15.22 ± 6.68, Pre-professional—5.5 ± 5.15; Males and Females	Classical ballet	Motion capture	Développé arabesque with and without elevé in CP1	Elite versus pre-professional	Moderate to serious
Gontijo et al. [57]	Age: 27 ± 8; Years of training: 18 ± 8; H/w: 4 ± 2 classes per week (no hours)	Classical ballet	Motion capture	Demi-plié & grand-plié in CP1	–	Moderate
Hackney et al. [58]	Age: 20.89 ± 2.93; Years of training: at least 5; Female	Classical ballet	Motion capture	Échappé sauté from CP1 to CP2 to CP1	–	Low to moderate
Tanabe et al. [59]	Pre-professional; Age: 24.1 ± 5; Years of training: 14.4 ± 3.6; Female	Classical ballet	Video analysis; EMG: Gm, RF, SAR, VL, BF, SM, MGAS, LGAS, SOL, FIB, TA, EDL, FHB	CP1 to CP6 & elevé	–	Low to moderate
Tanabe et al. [60]	Pre-professional; Age: 22.78 ± 4.68; Years of training: 11.56 ± 4.8; Female	Classical ballet	Video analysis; Force plates	Elevé	Dancers versus non-dancers	Low to moderate
Lin et al. [61]	Elite and Novices; Age: Superior experience—18.2 ± 1; Experienced—18.3 ± 5.7, Novice—12.3 ± 1.6; Years of training: Superior experience—9.8 ± 1.7, Experienced—8.6 ± 4.9, Novice—3.3 ± 1.7; H/w: Novices—1.5-3 h, Advanced—at least 3 h; Female	Classical ballet	Motion capture; Force plates	Retiré passé in CP5	Elite versus Novice	Moderate
Fong Yan et al. [62]	Pre-professional; Age: 25 ± 5.9; Female	Classical ballet	Motion capture; Force plates	Sauté in CP2	Barefoot versus jazz shoes	Low to moderate
Lin et al. [63]	Pre-professional; Age: Injured—19 ± 2, non-injured—17.7 ± 2.6; Years of training: at least 5; Female	Classical ballet	Motion capture; Force plates; EMG: FIB, MGAS, TA	Grand-plié in CP1	Injured versus non-injured	Moderate to serious
Lin et al. [64]	Elite and Novices; Age: Novices—12 ± 1.91, Advanced—17.77 ± 3.39; Years of training: Novices—3.23 ± 1.69, Advance—8.69 ± 3.3; H/w: Novices—1.5-3 h, Advanced-at least 3 h; Female	Classical ballet	Motion capture	En dehors pirouette in CP4	Elite versus Novice	Moderate to serious
Torrents et al. [65]	Pre-professional; Age: F—28 ± 12.7, M—31 ± 9.9; Years of training: at least 5; Males and Females	Contemporary dance	Motion capture	Tour en dehors, brisé volé en arrière en tournant, arabesque penchée	–	Low to moderate
Kiefer et al. [66]	Elite; Age: 23.59 ± 3.99; Males and Females	Classical ballet	Force plates	Demi-plié & elevé	Elite versus non-dancers	Low to moderate
Wyon et al. [17]	Pre-professional; Age: 20 ± 1.74; Female	Contemporary dance	Inertial sensors	Grand-jeté	–	RCT High Risk
Lobo da Costa et al. [67]	Pre-professional; Age: 18.4 ± 2.8; Years of training: at least 7; Female	Classical ballet	Force plates	Attitude devant, derrière & a la second	Ballet shoes versus barefoot	Low to moderate
Lee et al. [68]	Age: 19.73 ± 2.41; Years of training: at least 7; Female	Classical ballet	Motion capture; Force plates; EMG: FIB, TA, MGAS (both legs), VM, VL, AD, BF (dominant leg)	Sissonne fermée in CP5	Injured versus non-injured	Moderate
Pearson and Whitaker [69]	Pre-professional; Age: 19.63 ± 1.06; Years of training: At least 2 in pointe shoes; Female	Classical ballet	Force plates	Demi-pointe in CP1	Dancers with different shoes	Low to moderate
Shippen et al. [70]	Pre-professional; Age: 23; Female	Contemporary dance	Motion capture; Force plates	Contemporary sequence	–	Moderate
Bronner [71]	Elite and pre-professional and Novices; Age: Elite—24.9 ± 1, Intermediate—19.6 ± 0.5, Novice—19.8 ± 0.5; Years of training: Elite—13.3 ± 1.9, Intermediate—11.7 ± 1.1, Novice—6.1 ± 1.6; Males and Females	Classical ballet	Motion capture	Développé arabesque in CP1	Elite versus Pre-professional versus Novices	Moderate
Krasnow et al. [72]	Elite and pre-professional and Novices; Age: 30.0 ± 13; Years of training: 13.9 ± 13.3; Female	Classical ballet and modern dance	Motion capture; Force plates	Grand battement in CP1	Elite versus pre-professional versus novices	Moderate to serious
Charbonnier et al. [73]	Pre-professional and Elite; Age: 25.36; Years of training: at least 10; H/w: at least 12; Female	Classical ballet and modern dance	Motion capture	Arabesque, développé devant, développé a la seconde, grand écart facial, grand écart lateral & grand plié	–	Low to moderate
Lin et al. [74]	Pre-professional; Age: Injured—19.7 ± 2.4, Non-injured—18.8 ± 3.1; Years of training: at least 7; Female	Classical ballet	Motion capture; Force plates	CP1 and CP5	Injured versus non-injured versus non-dancers	Low to moderate
Walter et al. [75]	Pre-professional; Age: 19.94 ± 1.16; Years of training: 14.17 ± 2.92; H/w: 22.97 ± 8.41; Female	Classical ballet	Force plates	Assemblé in CP5	Flat shoes versus Pointe shoes	Low to moderate
Hackney et al. [76]	Pre-professional; Age: 22.72 ± 2.63; Female	Classical ballet	Motion capture	Grand-jeté	–	Low to moderate
Hackney et al. [76]	Pre-professional; Age: 21.31 ± 2.06; Female	Classical ballet	Video analysis; Force plates	Grand-jeté	–	Low to moderate
Hackney et al. [77]	Pre-professional; Age: 22.72 ± 2.63; Years of training: at least 5; Female	Classical ballet	Video analysis; Force plates	Sauté in CP1	–	Low to moderate
Bronner and Ojofeitimi [78]	Pre-professional; Age: 20.76 ± 2.46; Years of training: 10.74 ± 4.50; Males and Females	Contemporary dance	Motion capture	Grand battement devant, derrière & a la second in CP1	–	Low to moderate
Kulig et al. [79]	Pre-professional; Age: 18.9 ± 1.2; Years of training: 8.9 + 5.1; Males and Females	Classical ballet	Motion capture; Force plates	Saut de chat	–	Serious
Golomer et al. [80]	Elite; Age: Dancers—19 ± 1.6, non-dancers—19 ± 1.3; Years of training: at least 10; H/w: 35; Female	Classical ballet	Seesaw platform; Force plates	Postural sway in one leg	Elite versus non-dancers	Low to moderate
Imura et al. [81]	Pre-professional; Age: 27.7 ± 1.7; Years of training: 20.6 ± 3.2; Female	Classical ballet	Motion capture; Force plates	Fouetté turns	–	Low to moderate
Golomer et al. [82]	Elite; Age: 19 ± 2; Female	Classical ballet	Motion capture	Pirouette in CP4	–	Low to moderate
Golomer et al. [83]	Elite; Age: 19.6 ± 1.3; Female	Classical ballet	Motion capture	Pirouette in CP4	Elite versus non-dancers	Moderate
Imura et al. [84]	Pre-professional; Age: 27.7 ± 1.7; Years of training: 20.6 ± 3.2; Female	Classical ballet	Motion capture; Force plates	Fouetté turns	–	Low to moderate
Chockley [85]	Pre-professional; Female	Classical ballet	Force plates	Sauté in CP1	–	Moderate
Couillandre et al. [86]	Elite; Age: 31 ± 9; Female	Classical ballet	EMG: VL, BF, TA, SOL; Inertial Sensors	Demi-plié & sauté in CP1	–	Moderate
Golomer [87]	Pre-professional; Age: 19 ± 1.5; Female	Classical ballet	Motion capture	Pirouette in CP4	Dancers versus non-dancers	Low to moderate
Lepelley et al. [88]	Pre-professional and Elite; Males and Females	Classical ballet and modern dance	Motion capture; EMG: ES, GM, RA, P, BF, RF, VL, LGAS, SOL	Battement jeté	–	Low to moderate
Bronner and Ojofeitimi [89]	Elite; Age: F—30.7 ± 6.4, M—26.7 ± 4.9; Years of training: F—22.2 ± 6.1, M—14.2 ± 3.7; Males and females	Classical ballet and modern dance	Motion capture	Retiré passé in CP1	Male versus Female	Moderate to serious
Lin et al. [90]	Pre-professional; Age: 19.15 ± 1.9; Years of training: 11.37 ± 3.9	Classical ballet	Motion capture; Force plates	Relevé in CP1	–	Low
Thullier and Moufti [91]	Elite	Classical ballet	Motion capture	Rond de jambé	Elite versus non-dancers	Low
Golomer and Dupui [92]	Elite; Age: F—23.3 ± 6.7, M—24.1 ± 1.5, Untr. F—19.7 ± 2.6, Untr. M—24.3 ± 3; Males and Females	Classical ballet	Seesaw platform; Inertial sensors	Postural sway	Elite versus non-dancers	Serious
Golomer et al. [93]	Elite; Age: Dancers—23.8 ± 2.2, non-dancers—18.8 ± 3.5; Males	Classical ballet	Seesaw platform; Inertial sensors	Postural sway	Elite versus non-dancers	Serious
Golomer et al. [94]	Elite and Novices; Age: Adults—23.8 ± 2.2, Adolescents—18.1 ± 0.9, Novices—11.6 ± 1.3; Males	Classical ballet	Seesaw platform; Inertial sensors	Postural sway	Elite versus Novice	Serious
Golomer et al. [95]	Elite and Novices; Age: Elite—17.4 ± 1.1, Novices—11.9 ± 1.1, Acrobats elite—18.1 ± .1, Acrobats novices—12.5 ± 1.5; Female	Classical ballet and acrobats	Seesaw platform; Inertial sensors	Postural sway	Elite versus acrobats versus Novice	Serious
Trepman et al. [96]	Elite; Age: 33 ± 9; Years of training: 24 ± 10; H/w: 32 ± 7; Female	Classical ballet and modern dance	Video analysis; EMG: GM, BF, AD, VL, VM, TA, MGAS, LGAS	Demi-plié in CP1	Ballet versus modern	Low to moderate

Abbreviations: *EMG* electromyography, *ES* erector spinae, *RA* rectus abdominis, *GM* gluteus maximus, *Gm* gluteus medius, *SAR* sartorius, *BF* biceps femoris, *SEM* semitendinosus, *SM* semimembranosus, *ADL* adductor longus, *AD* adductors, *P* psoas, *RF* rectus femoris, *VL* vastus lateralis, *VM* vastus medialis, *VMO* vastus medialis obliquus, *LGAS* lateral gastrocnemius, *MGAS* medial gastrocnemius, *SOL* soleus, *TA* tibialis anterior, *FIB* fibularis longus, *EDL* extensor digitorum longus, *FHB* flexor hallucis brevis, *CP* classical ballet feet position (varying from 1 to 6)

The USA was observed to be the leading country of publications (26 articles), followed by France (11 articles), Australia (10 articles), Japan (8 articles), Taiwan (7 articles), UK (5 articles), Brazil, and Poland with 3 articles each country, Switzerland (2 articles), Colombia, Canada, Spain, Czech Republic, and Israel with 1 article per country.

**Fig. 2:**
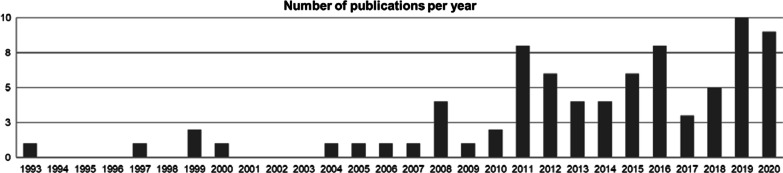
Yearly publications regarding studies of motor behavior in ballet (1993–2020)

Ballet research has increased in the past decade (Fig. 2). Between the years of 1993 and 2004, there were six publications regarding motor behavior in ballet, although numerous articles were found associating ballet to injury and rehabilitation processes.

### Category of Dance and Level of Expertise

Regarding the 80 articles included in the present systematic review, 60 studies have analyzed participants specifically from classical ballet; 14 have combined participants from classical ballet and modern dance; and 6 studies have analyzed participants from contemporary dance.

Thirty-nine studies analyzed and described ballet movements, without running any sort of comparisons between groups of participants regarding experimental conditions. These studies were divided as: (i) 25 studies with participants from classical ballet; (ii) 9 studies with participants from modern dance; and (iii) 5 studies with participants from contemporary dance. Concerning the participants' level of expertise, 11 out of the 39 studies recruited elite dancers as participants, 22 studies recruited pre-professionals, and 4 had elite dancers and pre-professionals within the same study (but without comparisons between levels of expertise). Two studies did not mention the level of expertise.

Forty-one studies have compared groups of the experimental design, with 14 studies comparing dancers to non-dancers (10 studies compared elite dancers to non-dancers), 5 compared elite to novices, 3 studies compared elite to pre-professionals to novices, and 1 study compared elite to pre-professionals. Six studies compared males to females. Four studies compared injured dancers to non-injured (one study did not mention the level of expertise but also compared injured to non-injured). According to the category of dance, 2 studies compared classical ballet to modern dance. Regarding practice conditions, 3 studies compared different types of shoes and 2 studies compared the condition of barefoot to wearing shoes. The remaining studies compared different groups under different experimental conditions. Twenty studies analyzed elite dancers, 19 analyzed pre-professionals, and 7 analyzed novices, considering that some of the studies combined different levels of expertise without comparing them, yet analyzing other variables, such as gender and different tasks. Only 1 study compared elite dancers with non-dancers and acrobats.

### Demographic Information

Three studies did not provide demographic information regarding participants’ age, years of practice, and hours of weekly training. Only 16 studies have provided all demographic information. Fifty-two out of 80 studies had only female participants, 22 had both males and females, 2 had only males, and 4 studies did not mention participants’ sex (Table 1).

### Sensing Technology

Forty-four studies used two or more types of technology to collect data, showing that 26 studies combined kinematic with kinetic analysis, 4 studies combined kinematic and kinetic analysis with EMG, 2 studies combined kinematic and kinetic analysis with inertial sensors, 4 studies combined kinematic analysis with EMG, 2 studies combined kinetic analysis with EMG, 5 studies combined kinematic analysis with inertial sensors, and only 1 study combined EMG with inertial sensors. The other 36 studies used only one type of technology to collect data, showing that 23 studies performed kinematic analysis (all used motion capture technique), 10 studies performed kinetic analysis (all used force plates), and 3 studies used inertial sensors only (Table 1). Overall, 64 studies performed kinematic analysis (49 studies used motion capture as technique), whereas 45 studies performed kinetic analysis (42 studies used force plates as technique). Twelve studies used inertial sensors as technique, and only 11 studies used EMG.

### Classical Ballet Movements Evaluated

In this systematic review, a total of 29 different ballet movements were analyzed within the selected articles (Table 1). The ballet movement with the most frequency of analysis was the *sauté* (15 studies). The second most studied movements were the *grand-jeté* and *saut de chat* (12 studies each)*.* Postural sway was analyzed in 9 studies, followed by the movement *demi-plié* and *en dehors pirouette* (8 studies each). Six studies analyzed the *grand-plié* movement. Static ballet feet positions and turnout of the hips were analyzed in 6 studies, and 7 other studies analyzed the *elevé* movement. Five studies analyzed the *arabesque* movement, and 4 studies analyzed the *relevé* movement. Three studies analyzed the *retiré passé* movement. Only 1 study analyzed upper limb ballet movements in a sequence of *port de bras*. Seventeen remaining movements were studied only once or twice, while the full list can be assessed in Table 1.

### Relationship Between Evaluated Ballet Movements and Sensing Technologies

Only 4 studies analyzed kinematics, kinetics, and EMG as protocol, and the selected movements were *grand-plié*, *relevé*, *sissonne fermée*, *arabesque*, and *cou-de-pied derrière* with *demi-plié* to *arabesque*.

Electromyography was analyzed in the following movements: *demi-plié* (3), *grand-plié* (1), *sauté* (2), 6 ballet positions (2), *elevé* (2), *relevé* (1), *arabesque* (2), *sissonne fermée* (1), and *battement jeté* (1).

Research that combined kinematic and kinetic analyses has studied the following ballet movements: postural sway (5), *saut de chat* (5), *grand-jeté* (4), *en dehors pirouette* (3), *sauté* (3), *relevé* (2), *fouetté* turns (2), *entrelacé* (1), *ballonné* (1), *assemblé dessus* (1), *bourrés* (1), *demi-plié* (1), *retiré passé* (1), *elevé* (1), contemporary sequence (1), *grand battement* (1), feet position (1).

Regarding the studies that only used one type of technology, 23 studies used motion capture systems to analyze kinematic variables of ballet movements such as *demi-plié* (4), *grand-plié* (3), *sauté* and *échappé sauté* (3), turnout of hips (3), *elevé* (2), *grand-jeté* (1) *battement fondu* (1), *ballonné* (1), *sissonne fondu* (1), *arabesque* (4), *en dehors pirouette* (5), *brisé volé* (1), *développé* (3), *grand battement* (1), whole body rotation (2), *retiré passé* (1), and *rond de jambé* (1). Ten studies only used force plates to analyze kinetics of ballet movements such as *grand-jeté* (1), *sauté* (2), *grand-plié* (1), *retiré passé* (1), *elevé* (2), *attitude* (1), *assemblé* (1), and postural sway (3). Three studies only used inertial sensors to analyze ballet movements such as *grand-jeté* (1), upper limb ballet postures (1), postural sway (1), and *cou-de-pied* with *fondu* (1).

### Relationship Between Motor Behavior and Brain Functional Analysis

Four studies were included regarding motor behavior approach with brain functional analysis. Those studies were performed by the same group of researchers [80, 82, 83, 87]. The authors have studied visual imagery and spatial context in combination with a motor control approach in the *pirouette* ballet movement. Visual imagery was assessed by the Vividness of Movement Imagery Questionnaire (VMIQ), and the authors evolved their research throughout the years, studying then the right hemisphere in visual regulation of complex equilibrium, since their previous research showed the influences of visual cues in the postural sway of ballet dancers.

## Discussion

In order to increase the scientific knowledge associated with the performance of ballet movements, the aim of this systematic review was to describe the technologies and devices used in data capture to analyze human performance and motor behavior of ballet movements. This review outlines the category of analyzed ballet movements in combination with sensing technology.

Classical ballet has a large lexicon of specific movements; consequently, this research field is still emerging. We found that only 29 ballet movements have been analyzed regarding motor behavior approach, which means that a baseline of data is being created in order to evolve to more complex movements.

Regarding the category of dance, most of the selected studies are in the classical ballet field [20–22, 24, 25, 28, 30–33, 35–41, 44–49, 51, 52, 54, 56–64, 66–69, 71, 74–77, 79–87, 90–95], although contemporary and modern dance became more popular recently [17, 19, 23, 26, 27, 29, 34, 42, 43, 50, 53, 55, 65, 70, 72, 73, 78, 88, 89, 96], probably because those categories of dance are offered in the curriculum of several colleges, since 22 out of 80 studies in this systematic review described participants as college dancers. Those participants were regarded as pre-professionals.

While disparities in skill levels were recognized between elite dancers and novices, mostly reporting that elite dancers have more effective and refined strategies regarding motor behavior and human performance (i.e., GFR, limb symmetry, muscle co-activation and so on), it is important to reach consensus in what is considered an elite dancer, as the definition of this category of dancers was found to be arbitrary in the evaluated studies [20, 21, 31, 33, 36, 50, 56, 61, 64, 71–73, 80, 88, 94, 95]. Number of years of practice, hours of training per week and professional career in ballet may be accurate factors to consider a professional dancer as an elite dancer. In other words, it is reasonable to think that elite dancers display higher performance in ballet movements than novices; however, it is important to establish a definition of what may be considered to be an elite dancer. Nonetheless, most of the studies included in the present systematic review had pre-professional dancers as participants, which allowed the understanding of movement pattern, although not representing the supremacy of the elite ballerina body. Study design in the published articles using pre-professional dancers should be redone with elite dancers as a follow up.

In effect, ballet research remains a field of interest in universities, mainly in graduate programs, and we found that only 28 out of 80 studies had some sort of funding or grants [20, 24, 25, 27–29, 31, 32, 38, 41–43, 46, 48–50, 55, 59, 63–65, 73, 80, 82, 83, 87, 89, 96].

Kinematic and kinetic analyses have been the prevalent techniques, having motion capture systems and force plates as the prevalent measurement tools, respectively. Our results reveal a lack of consensus in the research protocol regarding the experimental design, since several studies arbitrarily selected the movements but did not follow up with different tools to complement and improve data reliability. Combining two or more measurement tools may be paramount to optimize data collection and increase data reliability.

One limitation of the research studies so far is concerning the elements involved in motor coordination of ballet movements. For instance, only one study has analyzed upper limb movements of classical ballet [51]. Despite accepting a higher relevance of the lower limbs in the performance of ballet movements, upper limbs may also have a significant contribution to increase balance and movement fluidity, as we have found that postural sway plays an important role in motor behavior of ballet movements [28, 41, 44, 52, 80, 92–95]. Therefore, this gap could be suggested as an issue for further research, regarding coordination and the formation of motor synergies during the learning process and performance of ballet movements. For instance, ballet movements directly involving the neck and head, such as specific techniques to perform several revolutions in *pirouettes*, have not been studied yet. Variables such as movement speed, accuracy, and precision can be measured through motor behavior tools, also in conjunction with upper limb and postural data collection.

Differences in sex regarding motor behavior are well studied in the literature, and assumptions of sex differences have also been made in ballet research. Only 4 out of 80 studies in this systematic review actually made comparisons between males and females [21, 48, 52, 89]. This is a topic for future research regarding motor behavior and human performance in ballet.

The involvement of neuroscience in dance research has evolved in the past decade. Numerous studies combined imagery techniques and technology such as MRI and electroencephalography (EEG) [8, 97–99], as well as the mirror neuron system [100, 101], in order to understand the neurophysiology of ballet movements. However, just a few of those studies aimed to analyze brain–motor behavior connection, such as the studies included in this systematic review [80, 82, 83, 87]. It is of interest in ballet research to increase the knowledge regarding muscle–brain connection to better understand motor behavior and thresholds that distinguish levels of expertise. Perhaps this is the next obvious area of exploration.

The studies in this systematic review provide rich knowledge about the kinematics and kinetics of ballet movements. It is evident that researchers know more about ballet today than they knew in previous decades. Evidence has been built in ballet research regarding knowledge about motor behavior in dance, possibly allowing professional ballet companies and schools to better design ballet trainings in order to optimize human performance. Additionally, current findings in ballet research provide scientists with knowledge to pave the pathway for future and more complex data collection involving motor coordination, synergies, and brain activation. However, questions regarding the threshold that distinguishes novices from elite dancers remain unanswered. Although this review did not aim to evaluate clinical applications of ballet movements, the findings suggest that several ballet movements may be elected as rehabilitation techniques for protocol design. Conclusions in the literature are often found as suggestions to elaborate and improve training in order to both enhance performance and prevent injuries, as well as to, in some cases, perform specific dance movements as protocols for physical rehabilitation of non-dancers.

## Conclusion

This review highlighted the sensing technologies used to collect data of ballet movements. The findings represent an overview of the interests in motor behavior analysis regarding classical ballet movements. Studies in this review varied greatly considering study design and specific intervention characteristics. There is a broad collection of studies reporting motor behavior of several ballet movements with elite dancers, pre-professionals, and novices, in classical ballet, modern and contemporary dance. Technology is constantly evolving, and researchers are allowed to use modern tools to answer old questions about the mystery between art and sport that is present in classical ballet. The future of ballet research is promising, and it is exciting to foresee the upcoming results of a motor behavior approach to evaluate classical ballet.

## Data Availability

Not applicable.

## Abbreviations

AD: Adductors
ADL: Adductor longus
BF: Biceps femoris
CP: Classical ballet feet position
EDL: Extensor digitorum longus
EEG: Electroencephalograph
EMG: Electromyography
ES: Erector spinae
FHB: Flexor hallucis brevis
FIB: Fibularis longus
GM: Gluteus maximus
Gm: Gluteus medius
GRF: Ground force reaction
LGAS: Lateral gastrocnemius
MGAS: Medial gastrocnemius
MRI: Magnetic resonance imaging
non-RCT: Non-randomized controlled trial
P: Psoas
RA: Rectus abdominis
RCT: Randomized controlled trial
RF: Rectus femoris
SAR: Sartorius
SEM: Semitendinosus
sEMG: Surface electromyography
SM: Semimembranosus
SOL: Soleus
TA: Tibialis anterior
VL: Vastus lateralis
VM: Vastus medialis
VMIQ: Vividness of Movement Imagery Questionnaire
VMO: Vastus medialis obliquus
